# Malaria treatment failures after artemisinin-based therapy in three expatriates: could improved manufacturer information help to decrease the risk of treatment failure ?

**DOI:** 10.1186/1475-2875-5-81

**Published:** 2006-10-04

**Authors:** Yves Jackson, François Chappuis, Louis Loutan, Walter Taylor

**Affiliations:** 1 Travel and Migration Medicine Unit, Geneva University Hospital, Rue Micheli-du-Crest 24, 1211 Geneva 14, Switzerland; 2 UNICEF/UNDP/World Bank/WHO Special Programme for Research and Training in Tropical Diseases, World Health Organisation, Av. Appia 20, Geneva, Switzerland

## Abstract

**Background:**

Artemisinin-containing therapies are highly effective against *Plasmodium falciparum *malaria. Insufficient numbers of tablets and inadequate package inserts result in sub-optimal dosing and possible treatment failure. This study reports the case of three, non-immune, expatriate workers with *P*. *falciparum *acquired in Africa, who failed to respond to artemisinin-based therapy. Sub-therapeutic dosing in accordance with the manufacturers' recommendations was the probable cause.

**Method:**

Manufacturers information and drug content included in twenty-five artemisinin-containing specialities were reviewed.

**Results:**

A substantial number of manufacturers do not follow current WHO recommendations regarding treatment duration and doses.

**Conclusion:**

This study shows that drug packaging and their inserts should be improved.

## Background

Artemisinin derivatives, such as artesunate, artemether and dihydroartemisinin, are the most parasiticidal of all the antimalarial drugs against *Plasmodium falciparum *[1]. Their pharmacokinetic (rapid absorption, short half life ~1 hour) and pharmacodynamic (high parasite reduction ratio) properties mitigate against the development of resistance. *In vivo *resistance to this drug class has not yet been described [2]. However, several challenges remain, including the limited availability of GMP quality drugs, the emergence of fake drugs, and their optimal deployment in malaria-endemic countries. Artemisinin-based drugs are easily available in several malaria-endemic countries and are sold as mono- or combination therapies. They are manufactured in several countries, packaged differently and contain package inserts of varying quality and accuracy. WHO currently recommends malaria-endemic countries to use three days of an artemisinin-based combination treatment (ACT) e.g. artemether/lumefantrine (A/L), artesunate (4 mg/kg/day) plus an effective, longer acting partner drug, such as amodiaquine or mefloquine [3]. If used alone, seven days of treatment of an artemisinin derivative is necessary because shorter courses result in unacceptably high rates of recrudescence. Most recently, WHO has called drug manufacturers to switch the production of artemisinin monotherapies towards combined therapies because of the risk of developing drug resistance. Artesunate monotherapy of ≤ 5 days duration in South-East Asian and African patients is associated with a failure rate of up to 50% and the four doses regimen of artemether/lumefantrine has resulted in failure rates of between 15 to 18% in African children, Thai adults and non immune travellers [4-11]. Recrudescent falciparum malaria is associated with a risk of developing severe malaria, especially in non-immune individuals, such as travellers or expatriate workers.

Probable recrudescent *P. falciparum *malaria was recently diagnosed in three non-immune Europeans at the Geneva University Hospital. These three patients were initially treated in sub-Saharan Africa with artemisinin-based drugs. This motivated a thorough examination of package inserts of artemisinin-based drugs available in Africa. The potential association between treatment failure and inadequate information on drug leaflets are discussed in this study.

### Case report 1

Patient 1 was a 27 year old Swiss female working in Sudan for an international NGO. She was not taking antimalarial chemoprophylaxis. She developed a febrile illness that was diagnosed locally as malaria by positive microscopic examination (species and parasitaemia unknown) and Paracheck™, a rapid diagnostic test that detects *P. falciparum*-specific histidine-rich II protein. By inference, she was infected with *P*.*falciparum *either alone or mixed with another *Plasmodium *species. She self-prescribed oral artemether, obtaining the drug from the NGOs pharmacy. She did not recall the dose of artemether, but she reported full compliance with the five days course contained in the package. She improved on this treatment, but four days after stopping artemether, she developed fever again and had a positive malaria blood smear (species and parasitaemia unknown) consistent with a recrudescence of her primary infection. She was retreated with the same five days regimen with good effect. Some three and a half weeks later, she suffered a recurrence of fever associated with a positive malaria smear (species and parasitaemia unknown) suggesting either a recrudescence of her initial infection or a new infection. PCR genotyping was unavailable to differentiate her recurrent illness. She received artemether for seven days in combination with a single dose (three tablets) of sulfadoxine/pyrimethamine and had no further episodes of malaria while in Sudan. On review at the Geneva Hospital six weeks after her last febrile episode, she was well and afebrile.

### Case report 2

Patient 2 was a 35 years old Swiss woman working in rural Côte d'Ivoire for another international NGO. She was not on malaria prophylaxis. She developed fever and was found with a positive blood slide for malaria. Although the species was not specified, *P*. *falciparum *was the likely diagnosis given the high prevalence of *P*. *falciparum *locally. However, a mixed infection cannot be formerly ruled out. She was treated at a local clinic with a single dose of intramuscular quinine in combination with the four doses regimen (four tablets × 4 over 48 hours) of artemether/lumefantrine (A/L); the latter is marketed as CoArtem^® ^in malaria endemic countries. She made an uneventful recovery and then started daily chloroquine/proguanil (Savarine^®^) as prophylaxis. Twelve days after completing her A/L, she presented at Geneva Hospital with fever and had a positive malaria slide, with a *P*.*falciparum *asexual parasitaemia of 4,000/μL. She was treated successfully with three days of atovaquone/proguanil, remaining afebrile and aparasitaemic during 28 days of follow up.

### Case report 3

Patient 3 was a 30 years old Swiss man who was working in Guinea Conakry. He took weekly mefloquine as prophylaxis and reported good compliance. However, he developed an acute febrile illness that was diagnosed as malaria by blood smear. He was unaware of the *Plasmodium *species but, considering the local malaria epidemiology, it was almost certainly *P*. *falciparum*. A local physician treated him with oral artesunate for three days and sulfadoxine/pyrimethamine, as a single dose of three tablets, with good effect. He did not recall the dose of artesunate but it was probably either 50 mg bid or 100 mg bid on the first day followed by 50 mg twice a day, consistent with the artesunate formulations likely to be available locally. However, 14 days after as the last dose of artesunate, he presented in Geneva with fever and his blood slide was positive for *P*.*falciparum *with an asexual parasitaemia of 190,000/μL, consistent with a probable recrudescence of his primary illness rather than a new infection (PCR unavailable). He was cured with the six doses regimen of artemether/lumefantrine.

In summary, these three non-immune expatriate workers suffered of probable *falciparum *malaria treatment failure after initial diagnosis and artemisinin-based treatment in Africa. It must be emphasized that the initial diagnosis could not be ascertained as malaria blood slides were not available for quality control. Considering the great variability in quality of diagnostic procedures in African laboratories, local diagnosis of malaria may not always be accurate.

All patients reported having completed their treatment without episode of vomiting. The early timings of their recurrent malaria are consistent with recrudescence, but in the absence of PCR genotyping the possibility of early reinfections cannot be definitively excluded. Similarly, in the absence of defined plasmatic drug levels, none of these patients' recurrences meets the WHO definition of drug resistance [12]. Sub-optimal drug dosing is the likely explanation for the treatment failures in all three patients. Patient 1 took artemether for an inadequate duration (five days only) and suffered recurrent malaria four days after completing her treatment. Patient 2 failed with the four dose regimen of artemether/lumefantrine, an expected finding in non-immunes [9]. Patient 3 had probably taken insufficient artesunate during the first three days. His total dose was probably either 400 mg (200 mg day 1, 100 mg days 2 & 3) or 300 (100 mg/d) mg. These doses translate to a mean of 1.9 and 1.4 mg/kg daily, respectively, and are less than half of the current recommendation of 4 mg/kg (~300 mg for a 70 kg man) of artesunate daily when used in combination. However, the failure to clear parasites following an adult dose of sulfadoxine/pyrimethamine (S/P) is consistent with S/P resistance.

Several artemisinin-containing therapies and their packaging were reviewed in order to assess the role of inadequate manufacturer information and insufficient drug content as possible causes of malaria treatment failure in sub-Saharan Africa.

## Methods

Packages of artemisinin-containing therapies currently sold in Africa were obtained from returning patients, WHO Roll Back Malaria, and the Internet. The list of twenty-five specialities presented here is not exhaustive (Table 1), but can reasonably be considered as representative of the current African market.

**Table 1:** Examples of artemisinin-based monotherapies and combinations currently sold in Africa for the treatment of uncomplicated falciparum malaria with summarized informations provided by the manufacturer on the accompanying package insert.

**Trade name**	**Drug substance/s**	**Tablet strength (mg)**	**Adult dose/s (mg/day)**	**Paediatric dose/s (mg/day)**	**Duration (days)**	**Tablets/package**	**Company**
***Combination products***							
Artekin – 02	dihydroartemisinin+ piperaquine	40/320	160/1280	7–10 y 80/640 11–15 y 60/480	2	8	Hualijian
Artekin	dihydroartemisinin+ piperaquine	40/320	160/1280	7–10 y 80/640 11–15 y 60/480	2	8	Holleykin pharmaceutical
Duo-cotecxin	dihydroartemisinin+ piperaquine	40/320	160/1280	7–10 y 80/640 11–15 60/480	2	8	Beijing Holley-Cotec
Coartem	artemether+ lumefantrine	20/120	D1 160/960, D2-3 80/480 or D1-3 160/960	5–15 kg 40/240 16–25 kg 80/480 26–35 kg 120/720	3	16–24	Novartis
Artequin	artesunate + mefloquine	200/250	200/250	NA	3	3 + 3	Mepha
Arsucam (infants and children)	artesunate + amodiaquine	50/153	NA	1–7 y 50/153 7–13 y 100/306	3 3	3 + 3 6 + 6	Sanofi-Aventis
Arsucam (adults)	artesunate + amodiaquine	50/153	200/612	NA	3	12 + 12	Sanofi-Aventis
Larimal	artesunate + amodiaquine	50/153	200/612	4 mg/kg (AS) + 10 mg/kg (AM)	3	12 + 12	Ipca
***Monotherapies***							
Artesunate tablets	artesunate	50	D1 200, D2-5 100	NA	5	6	Guilin pharmaceutical
Arinate	artesunate	100	D1 200, D2-5 100	D1 3.2 mg/kg, D2-5 1.6 mg/kg	5	6	Dafra
Arsumax	artesunate	50	D1 200, D2-5 100	D1 4 mg/kg, D2-5 2 mg/kg	5	12	Sanofi-Aventis
Cotexin	artesunate	60	D1 120, D2-5/7 60	NA	5–7	NA	Cotec
Artesunate injection	artesunate	60	NA	NA	NA	NA	Guilin pharmaceutical
Arte-biosorp	artesunate	75	300	NA	7	NA	Hovid
Artenex	artesunate	NA	D1 4tab, D2-5 2tab	D1 3.2 mg/kg, D2-5 2 mg/kg	5	12	Kinapharma
Malarin 200	artesunate	200	D1 400, D2-5 200	D1 4 mg/kg, D2-5 2 mg/kg (infants) D1 200, D2-5 100 (children)	5	6	tri-health
Plasmotrim 200	artesunate	200	D1 400, D2-5 200	NA	5	6	Mepha
Plasmotrim 50	artesunate	50	NA	D1 10 mg/kg, D2-5 5 mg/kg	5	12	Mepha
Gvither forte kit injection	artemether	80	D1 240, D2-5 80	D1 4 mg/kg, D2-5 2 mg/kg	5	6	Gvs labs
Gvither forte tablets	artemether	80	D1 240, D2-5 80	D1 4 mg/kg, D2-5 2 mg/kg	5	6	Gvs labs
Larither capsule	artemether	40	D1 4 mg/kg, D2-5 2 mg/kg	NA	7	6	Ipca
Capsulae artemetheri	artemether	40	D1 160, D2-5 80	NA	5–7	10	Kunming pharmaceutical
Arte-biosorp	artemisinin	75	150	NA	7	30	Hovid
Malaxin	dihydroartemisinin	60	D1 120, D2-5/7 60	NA	7	8	Cho dang pharmaceutical
Cotecxin	dihydroartemisinin	60	D1 120, D2-5/7 60	D1 60, D2-7 30	7	8	Jiaxing nanhu

Abbreviations: NA: information not available. D1,2,3: first, second, third day of treatment. Y: year. Tab: tablets

## Results

The majority (10/17) of manufacturers of artemisinin monotherapies recommend five days of treatment instead of the WHO recommended seven days. Only three drug packages had paediatric dosing and none had specific recommendations for non-immune patients e.g. that 5 days of treatment is likely to be inadequate. Some packages contained less tablets than indicated in the package insert. The package inserts included in combination treatments were generally better, with doses both for children (7/8) and adults and with sufficient tablets to complete a therapeutic course. Of note was the use of the lower dose of mefloquine (15 mg/kg) rather than the higher dose (25 mg/kg) that is used in multidrug resistant areas of SE Asia.

## Discussion

Sub-optimal antimalarial therapy results in increased morbidity and mortality of individuals living in malaria endemic areas, as well as travellers and expatriate workers [13,14]. Whilst well-established risk factors include poor compliance, fake drugs, limited training of drug vendors, high cost and drug resistance [15-17], little attention has focused on the number of packaged tablets and the quality of information in package inserts. Both are crucial elements in the successful treatment of malaria. Many patients in endemic countries buy antimalarial drugs without prior medical consultation from local markets, small shops, pharmacies and mobile street vendors. Most would probably assume that one drug package contains a therapeutic course. Travellers and field workers probably have the same assumptions as local residents. Clearly, drugs that are sold already packaged with insufficient tablets to make up a therapeutic course carry a risk of treatment failure, which is increased further, if not all of the packaged drugs are taken. Drugs at parasiticidal concentrations need to act on at least four asexual erythrocytic cycles to eliminate all asexual malaria parasites; thus, a minimum of six days of therapeutic drug concentrations is required [1]. Published data document failure rates of 50% with three days of artesunate in Africans, 50% with five days of artesunate in Vietnamese patients, and 18% with the for four doses regimen of artemether/lumefantrine in European travellers [5,7,9].

This study shows that a substantial number of artemisinin-based drugs currently sold in Africa do not respect current WHO recommendations in term of treatment duration in case of monotherapy and many lack appropriate information regarding children dosage. Packages containing fewer pills than indicated in the manufacturers leaflet were also found. Concerning the use of low dose of mefloquine (15 mg/kg) in combination treatment, this may not be a problem in the short term in Africa where mefloquine as monotherapy or combined with artesunate has shown high efficacy in recent trials. Nevertheless, great care should be taken in monitoring cure rate, as this lower dose may potentially favour the development of mefloquine resistance [18-20].

## Conclusion

Travellers, NGO and other workers should be aware not only of the hazards of contracting malaria, but also how to obtain optimal treatment. This is especially relevant for those who may be located in remote rural areas. Drug manufacturers should also play their part in reducing the risks of treatment failure. It is suggested that all currently available artemisinin-based monotherapies should be packaged for adults and children with a complete therapeutic course of seven days of treatment, that package inserts should be improved e.g. warning that a full course of treatment must be taken even if patients feel well. In view of recent WHO recommendations, manufacturers should shift their production in favour of combined therapies rather than monotherapy to avoid the development of resistance for what is often called the 'last effective weapon against *falciparum *malaria'. Written information should avoid proposing different doses by trying to differentiate patients based on their presumed malaria immune status, as this may be confusing both for patients and prescribers. Similarly, packaging for combinations should be explicit, easy to use and contain the requisite number of tablets. Travellers should be aware that artemether/lumefantrine is known as Riamet^® ^in temperate countries and CoArtem^® ^in malaria-endemic countries. Adult Riamet^® ^contains 24 tablets (six dose regimen) but adult CoArtem^® ^contains only 16 tablets (4 dose regimen). Setting international standards on package inserts might be a useful step that could be championed by a relevant international body. The training of local drug sellers and community health workers in malaria endemic countries is another strategy that could be adopted.

## Authors' contributions

YJ: collected and analysed data and wrote the manuscript

FC: clinically managed the patients and co-supervised the study and the writing of the manuscript

LL: co-supervised the study and revised the manuscript

WT: collected data and co-supervised the writing of the manuscript
